# Family-Centered Model and mHealth Through Early Intervention in Rural Populations: A Quasi-Experimental Study

**DOI:** 10.3390/children12020212

**Published:** 2025-02-11

**Authors:** Estíbaliz Jiménez-Arberas, Yara Casáis-Suárez, Sara Menéndez-Espina, Sergio Rodríguez Menéndez, Alba Fernández Méndez, José Antonio Prieto Saborit

**Affiliations:** Faculty Padre Ossó, University of Oviedo, 33008 Oviedo, Spain; estibaliz@facultadpadreosso.es (E.J.-A.);

**Keywords:** early intervention, telemedicine, child development, rural population, family

## Abstract

Background: Considering the challenges of Early Childhood Intervention (ECI), especially in rural areas with limited access to resources, it is essential to explore innovative strategies to address these barriers. Recent research highlights the use of mHealth to improve the accessibility and effectiveness of interventions. This study aims to evaluate the impact of mHealth interventions within the family-centered model on child development and familial quality of life, compared to the child-centered model. Methods: This study employs a quasi-experimental design with pre- and post-test measures across two control groups (control group 1: child-centered model and control group 2: family-centered model) and one experimental group (family-centered model with mHealth). The sample consisted of 55 children (11 girls and 44 boys) aged 0 to 6 years old with neurodevelopmental disorders that resided in rural areas from June to September 2023. For the assessment of the family unit, an ad hoc sociodemographic questionnaire, the Family Quality of Life Scale, the Family Confidence Scale, and the Family Outcomes Scale were used. Children were evaluated using the Pediatric Evaluation of Disability Inventory and the Battelle Developmental Inventory Screening Test. Results: Results revealed significant differences between the experimental group (EG) and the control groups. The EG, which received mHealth-based interventions within the family-centered model, demonstrated the greatest improvements in variables related to family quality of life and the child’s social functioning. Conclusions: mHealth appears to be a promising solution for improving access to early childhood intervention in rural areas, enhancing childhood development and a family’s quality of life. Strong therapeutic relationships, supported by interdisciplinary and individualized approaches, are key to maximizing its impact.

## 1. Introduction

Neurodevelopmental disorders are conditions characterized by impairments in various areas of functioning, such as cognitive, social, and academic abilities, which become evident in early childhood according to the American Psychiatric Association, Diagnostic and Statistical Manual of Mental Disorders (DSM-5) [1]. Recent studies reveal that 10% of the global pediatric population is diagnosed with one or more neurodevelopmental disorder (NDD), affecting various areas of functioning and limiting their activities of daily living (ADLs) [2].

One of the most active research areas in pediatrics is outcome measurement in this population, specifically within Early Childhood Intervention (ECI) [3,4]. These interventions can vary depending on the developmental context. Previous studies highlight limitations in rural areas due to restricted access to care resources or the impact of geographic conditions [5,6]. Moreover, the scarcity of specialized professionals or insufficient transport networks hinders intervention availability. Authors like Dew et al. [7] and Tolosana and Serrano [6] have reported restrictions in the participation of children with disabilities living in rural areas due to connectivity and transportation barriers.

The main challenge faced by ECI in many areas of Spain is the restrictions in the access to resources, since rural areas are far away from healthcare services, so in order to receive early childhood care treatment, families often require their own vehicle and time to travel. A challenge at this level is to achieve equity in access and provision of early care resources between rural and urban areas. Currently, in the Principality of Asturias, Early Childhood Care Units have been set up at the extremes of the territory as well as better internet connectivity. Therefore, this model proposes a possibility to reduce the displacements of families to the centers to receive treatments as well as a more continuous and direct attention that facilitates to carry out the guidelines and the generalization of learning to the real environment, a challenge for child development in all areas. The scientific literature underscores the urgent need to analyze and research new trends in ECI, considering environmental factors that may influence the intervention process and the children’s development [3,8].

Pons and Gelabert [9] advocated for interventions conducted in natural environments, where professionals provide services in the child’s usual settings, such as home or school, to empower caregivers and to facilitate the achievement of developmental milestones. This approach necessitates developing intervention strategies to overcome intrinsic contextual barriers. Addressing this need, information and communication technologies (ICT) have emerged as a viable option to ensure access to various health intervention services [10]. In this context, remote interventions, or teleinterventions, have gained traction, with mHealth defined as the use of mobile phones, wireless devices, and digital assistants to improve healthcare delivery [11].

In the field of occupational therapy, various models and approaches guide professional interventions in ECI, notably the child-centered model, rooted in Roger’s theory [12] that considers the child’s innate potential for development, with the professional supporting this process. Additionally, the family-centered treatment model [13,14] involves professionals working collaboratively with families, encouraging active roles through active listening and joint strategies to promote development in natural environments [15,16,17].

Angell et al. [18] emphasized the use of strategies based on this model as a way to generalize learning to real environments and empower families to support their children in daily activities. Recently, Jimenez-Arberas et al. [19], in a systematic review, concluded that service perception is influenced by the quality of the therapeutic relationship. Considering families’ values, beliefs, needs, and concerns creates a safe environment, increasing satisfaction, confidence, and competence—factors intrinsically linked to family quality of life [20], which is the ultimate goal of ECI.

From a global perspective, applying techniques or methods utilizing mHealth could be considered a solution to contextual barriers. This strategy fits within the family-centered model due to the active participation required from families and the opportunity for joint problem-solving between them and professionals to foster child development and improve the functioning and quality of life of the family unit [21,22,23].

Thus, the ultimate goal of this study is to evaluate the effectiveness of mHealth use within the family-centered model and assess its impact on development and family quality of life. To this end, a null and an alternative hypothesis are proposed:

**Null** **Hypothesis** **(H_0_).** *Interventions using mHealth within the family-centered model do not generate greater changes in family well-being compared to interventions based on the child-centered model, nor to those using the family-centered model implemented exclusively through in-person sessions.*

**Alternative** **Hypothesis** **(H_1_).** *Interventions using mHealth within the family-centered model generate greater changes in family well-being compared to in-person interventions, as well as compared to those based on the child-centered model interventions based on the child-centered model.*

## 2. Materials and Methods

### 2.1. Study Design

This study employed a quasi-experimental design with pre- and post-test measures across one experimental group and two control groups. The experimental group received a treatment based on the mHealth methodology, while the control groups received only in-person treatment. The use of two control groups allows for a comparison between the intervention that combines a family-centered model and mHealth methodology with two other distinct in-person interventions: one based on the family-centered model and the other on the child-centered model.

### 2.2. Participants

Participants were recruited through non-randomized convenience sampling from (1) previous research projects within the same organization and/or (2) interest generated through a dissemination plan targeting non-profit organizations identified as potential beneficiaries. Subsequently, contact was established with various families, who were informed about the study and asked for their voluntary participation.

All participants were aged between 0 and 6 years old, resided in rural Asturias, and had a diagnosis of neurodevelopmental disorder (NDD) according to the DSM-5 [1].

### 2.3. Measurement Intruments

The questionnaires were administered in two ways. All of the questionnaires listed below were digitally self-administered by the families, with the exception of the Battelle Developmental Inventory Screening test, which was administered by the therapist directly with the child using pen and paper.

Ad Hoc Sociodemographic Questionnaire for Children and Families: This questionnaire consisted of 44 questions, with 27 pertaining to demographic data and the child’s clinical history and 17 addressing family-related aspects such as parental education levels and the feasibility of reconciling work and caregiving responsibilities.

Family Quality of Life Scale (FAQOL): This scale [24,25], validated in Spanish by Verdugo et al. [26], assessed family quality of life through 41 items grouped into six factors: family interaction, parenting, health, safety, family resources, and support for individuals with disabilities. Each item was scored on a 5-point Likert scale. The internal consistency of the Spanish version was α = 0.96.

Family Confidence Scale (Con-Fam SCALE): Developed by McWilliam and García-Grau [27] and Subinas-Medina et al. [28], this tool measured family confidence through two subscales: Con-Fam CAN (α = 0.96), assessing caregivers’ perceived confidence in helping the child participate in daily routines, and Con-Fam CAF (α = 0.94), assessing self-perceived confidence in supporting oneself and one’s family. The scales comprised 20 and 18 Likert-type items (1–4), respectively.

Family Outcomes Survey (FOS): This survey [29,30] included 24 items measuring family knowledge and skills across five outcomes: understanding their child’s strengths, needs, and abilities; knowing their rights and advocating for their child; supporting their child’s development and learning; building support systems; and accessing the community. Items were rated on a 5-point Likert scale (nothing, a little, somewhat, almost, completely). The internal consistency was α = 0.90.

Pediatric Evaluation of Disability Inventory (PEDI): This instrument [29,31] assessed functional abilities in children aged 6 months to 7.5 years with physical and/or cognitive limitations. Functional activities were divided into three domains: self-care (197 items), mobility (20 items), and social function (20 items). Responses were dichotomous: 0 = unable, 1 = capable. The internal consistency of the Spanish version ranged from α = 0.89 to α = 0.98 across subscales.

Battelle Developmental Inventory Screening (BDI): This tool [32,33] evaluated key developmental domains in children aged 0–8 years. It comprised 100 items across five developmental areas, rated on a Likert scale (0–2). The internal consistency, measured through item-test correlations, ranged between α = 0.92 and α = 0.96 [34].

### 2.4. Procedure

A multidisciplinary clinical team was formed, consisting of a physiotherapist, a psychologist, a speech therapist, and an occupational therapist. Their work with the children and their families was organized over a 6-week period as follows:

- Week 1: Pre-test evaluation.
- Weeks 2–5: Intervention phase, with one session per week for each child.
- Week 6: Post-test re-evaluation using the same tools as in the initial assessment.

Following the pre-test evaluation, the specific areas for intervention were identified for each child. Based on the results obtained, different professionals were assigned to each case to ensure personalized and needs-oriented care. The intervention process for each group was structured as follows:

- Control Group 1 (CG1): Child-Centered Model. Weekly in-home sessions were conducted directly with the child by the assigned professional(s).
- Control Group 2 (CG2): Family-Centered Model. A biweekly in-home intervention plan was developed, involving the child and at least one caregiver. Between sessions, written support materials were provided to facilitate the generalization of strategies to the natural environment.
- Experimental Group (EG): Family-Centered Model with mHealth. This group followed a similar dynamic to CG2, with the exception that support materials were delivered in the form of videos and/or infographics created by the professional team tailored to the family’s and child’s specific needs. That is, the care was provided at the child’s own home together with their family in 45-min sessions by each professional who performed the intervention according to the child’s needs (occupational therapist, psychologist, speech therapist and physiotherapist) and the following week in the same time slot and day, the intervention was performed using the application by the family (the materials were created specifically for each child’s needs and by each professional). Additionally, an open communication channel was maintained to promptly address any doubts or concerns that the families had while implementing the provided strategies. (https://sway.cloud.microsoft/lZgBYGh5ooo5EGoL?ref=Link and https://www.youtube.com/playlist?list=PL98UjwLPeYPSQPEfQ85_IO5gPOHcmxtCB, accessed on 7 January 2025).

The materials hosted in the application consisted of videos specifically created to address the intervention needs of the children. These videos were designed in a way that allowed families to implement them comfortably at home while being supported by a professional—either in person or through the application to resolve any doubts. Additionally, infographics were developed to provide general guidelines for facilitating the transfer of intervention strategies to the home environment. Furthermore, publicly accessible supplementary materials were included to enhance family knowledge, such as a mapped directory of recommended community resources within the autonomous region. https://mapeoderecursos.inypemalivinglab.es/, accessed on 3 February 2025).

### 2.5. Ethical Considerations

The Research Ethics Committee for Medicinal Products of the Principality of Asturias approved the project titled “Outcome Measures in Natural Environment Interventions in Early Childhood Intervention” under the code CEImPA 2023.342.

### 2.6. Data Analysis

Initially, all variables, both pre-test and post-test measures, were subjected to a parametric analysis within each group to assess normality using the Shapiro–Wilk test (α = 0.05). For subsequent multivariate analyses, the assumption of homogeneity of variances was verified using Levene’s test (α = 0.05).

#### 2.6.1. Intra-Group Differences

A paired-samples *t*-test was conducted to analyze mean differences between pre-test and post-test measurements within each group (α = 0.05). For variables where normality assumptions were violated at either time point, the non-parametric Wilcoxon signed-rank test was used. Effect sizes were calculated for significant differences (*p* < 0.05). For contrasts using Student’s *t*-test, Cohen’s d was used to estimate effect size, while biserial rank correlation was applied for non-parametric contrasts.

#### 2.6.2. Between-Group Differences in Pre-Test and Post-Test

An independent-samples *t*-test was conducted using pre-test data to determine if variable levels were homogeneous across groups. The comparisons followed the quasi-experimental design: quasi-experiment 1 (comparison between CG1 and EG) and quasi-experiment 2 (comparison between CG2 and EG). The same procedure was applied to post-test data.

Student’s *t*-test was used when normality and homoscedasticity assumptions were met, while non-parametric tests, such as Mann–Whitney U and Welch’s *t*-test, were applied when these assumptions were violated. No correction for multiple comparisons was applied because many of the variables had not been previously studied in this population. This gives the study an exploratory nature, and post hoc corrections are highly conservative, which could reduce the ability to detect relevant effects of the treatment. Effect sizes were calculated using Cohen’s d for statistically significant differences.

## 3. Results

The final sample consisted of 55 children living in rural Asturias, of whom 20% (n = 11) were girls and 80% (n = 44) were boys. The mean age was 42.72 months (SD = 22.51).

The distribution across the three intervention groups was as follows:

- Control Group 1 (CG1): Comprising 42% of the final sample (n = 23), with a mean age of 57.63 months.
- Control Group 2 (CG2): Representing 30% of the participants (n = 17), with a mean age of 53.05 months.
- Experimental Group (EG): Comprising 27% of the sample (n = 15), with a mean age of 37.8 months.

In accordance with the objectives proposed in this study, regarding the assumption of normality, the results based on the pre-test period of the different groups indicated that GC1 does not meet this assumption for the variables PEDI mobility (W = 0.773; *p* < 0.01), FOS 1 (W = 0.032; *p* < 0.01), FOS 5 (W = 0.910; *p* < 0.05). In GC2, the assumption is violated for FOS 2 (W = 0.882; *p* < 0.05), FOS 3 (W = 0.802; *p* < 0.05), FOS 4 (W = 0.915; *p* < 0.05), and FOS 5 (W = 0.910; *p* < 0.05); and in GE for the variables FOS 1 (W = 0.857; *p* < 0.01), FOS 3 (W = 0.810; *p* < 0.05). In the post-test period, the assumption is violated in GC2 only for the variables Family Quality of Life (FQL) FN (W = 0.992; *p* < 0.05) and FOS 5 (W = 0.867; *p* < 0.05), and in GE for the variables BATTELLE (W = 0.858; *p* < 0.05) and FQL Total (W = 0.706; *p* < 0.01) (Appendix A Table A1)

Regarding the homogeneity assumption, violations were observed only between CG2 and EG in the variable PEDI Mobility, both in the pre-test (F = 12.593; *p* < 0.001) and in the post-test (F = 7.026; *p* < 0.013) (Appendix A Table A2).

In the *t*-test for mean differences between CG1 and EG, a statistically significant initial level was observed in the pre-test period for the Confidence Scale (CAST) (t = 2.041; *p* < 0.05; d = 0.714). In the post-test period, significant differences were identified in BATTELLE (w = 235.00; *p* < 0.05), with an increase in the mean for CG1 and a moderate effect size (d = 0.729) (Table 1).

Considering the variable of implementing interventions based on the family-centered model, with or without mHealth, the inter-group analyses showed differences between GC 2 and GE in both data collection periods. In the pre-test, there were differences in the variables BATTELLE (t = −2.534; *p* < 0.05), PEDI Mobility (t = −2.526; *p* < 0.05), FQL RF (t = 2.782; *p* < 0.01), FQL AIS (t = 2.150; *p* < 0.05). In the post-test, significant differences were observed in BATTELLE (t = 77.500; *p* < 0.05) and PEDI Mobility (t = −2.357; *p* < 0.05), with higher means in GE. Significant differences were also detected in FQL RF (t = 2.391; *p* < 0.05) and FOS 4 (t = 2.612; *p* < 0.015), both with higher means in GC 2 (Table 2).

Regarding intra-group differences between the pre- and post-test periods (Table 3), CG1 showed significant changes in the variables BATTELLE (w = 1.000; *p* < 0.01; d = −0.990), PEDI Mobility (w = 18.500; *p* < 0.01; d = −0.805), and FOS 1 (t = 3.154; *p* < 0.01; d = 0.688). In CG2, a significant change was identified in BATTELLE (t = −3.632; *p* < 0.05; d = −0.856). Finally, EG exhibited statistically significant changes with increases in the mean across the following variables: PEDI Social (t = −2.745; *p* < 0.01; d = −0.709), ConFam Total (t = −2.855; *p* < 0.05; d = −0.824), ConFam CAN (t = −2.780; *p* < 0.05; d = −0.802), and FOS 2 (t = −2.714; *p* < 0.05; d = −0.784).

The individual analysis of contrasts between both periods revealed that the EG, which received the intervention using mHealth within the family-centered model, showed the highest number of changes in family-related variables, as well as significant improvements in the child’s social functioning.

Similarly, CG1, which followed a child-centered intervention, displayed a higher mean in general development and mobility function. However, there was a decrease in the mean related to families’ understanding of their child’s strengths, needs, and abilities. For CG2, the only significant improvement observed was in the general development of the child.

## 4. Discussion

The primary objective of this study was to evaluate the effectiveness of using mHealth as a strategy within early childhood intervention and its impact on child development and family quality of life.

Scientific literature emphasizes the importance of providing effective early interventions for children with limitations in daily functioning [3,4]. A review by Kumar et al. [35] suggested that these practices not only enhance skill development but also promote participation and functionality across diverse contexts. Early interventions in pediatrics are, therefore, essential for addressing individual needs and maximizing developmental potential [36].

However, contextual factors such as geographic location and socioeconomic status can hinder access to and provision of care resources, thereby impacting treatment effectiveness [7,37]. These factors underscore the need to tailor interventions to the specific realities of each family to ensure their effectiveness and sustainability [6].

This study revealed differences in the effectiveness of interventions based on different theoretical models. Child-centered interventions showed positive effects on general development and mobility function. Recent studies support the use of this model to enhance the self-management of the child receiving the intervention [38,39]. However, a notable challenge was the decline in families’ ability to recognize their child’s strengths and abilities in everyday situations, such as participation in daily activities. This suggests that while these interventions are valuable, they may create a gap between professional care for the child and the support provided by caregivers [40]. Furthermore, the availability of accessible resources may influence the implementation and effectiveness of such interventions [41].

On the other hand, family-centered interventions demonstrated significant improvements in general child development [42]. A review by Jenkin et al. [43] highlighted various caregiver participation strategies during the intervention process, emphasizing the need to strengthen theoretical models to improve communication pathways between professionals and caregivers. This enhancement can foster trust and improve family quality of life [20,44].

A strong therapeutic bond [21,22,45] and active family participation [46,47,48] are critical for the success of these interventions. However, barriers such as caregiver resistance to change [20,48,49] and insufficient resources to sustain participation [41,50] may limit their effectiveness. In this study, the intervention timeframe may not have been sufficient to establish a strong therapeutic relationship. Such difficulties are particularly relevant in rural or low-resource settings, where access to services is limited, and families require greater flexibility and support for full participation [6,7,51].

Psychosocial and cultural factors also play a crucial role in the adoption and effectiveness of intervention models [19]. Cultural norms and family expectations shape how these strategies are perceived and adopted, highlighting the need for tailored approaches to ensure success [52,53].

In this context, mHealth has emerged as a promising strategy for delivering health interventions, particularly during and after the COVID-19 pandemic, and continues to evolve [18,54,55]. The results of this study demonstrate that mHealth-based interventions led to a greater number of significant changes between the pre- and post-test periods compared to other analyzed models. These changes impacted both the child’s functionality and family confidence and outcomes. Other studies report that remote interventions have expanded service reach in contexts with limited access and have enhanced active family participation, strengthening the child–professional–family triad [21,22,56], a key differentiator compared to the family-centered model without mHealth.

It is crucial to consider the limitations of mHealth, such as the need for adequate digital infrastructure, digital literacy among families, and challenges related to data privacy and security [57,58,59]. Despite these challenges, mHealth is increasingly recognized as a facilitator of continuous communication, enabling families to take a more active role in supporting their child [43,46,47,48].

### Limitations and Future Lines of Research

This study presents some limitations related to the use of mHealth. Firstly, the small sample size prevents establishing causal relationships and limits the generalization of results. Additionally, the entire sample was drawn from a single territory, the Principality of Asturias, which has unique sociodemographic characteristics that may not resemble other rural contexts. On the other hand, regarding the child’s assessment process, this study did not assess the level of digital education or technological accessibility, factors that could indirectly influence the child’s social functioning. Future studies using mHealth should consider these variables.

Moreover, the duration of the prescribed interventions may have been insufficient to achieve certain objectives or to perceive significant differences between the pre-test and post-test periods. In addition, the mean age of the experimental group is significantly lower than that of control group 1 and control group 2, this could be a bias in our study because intervention at younger ages could cause greater clinical improvement.

In future studies, the degree of NDD that the children have will be taken into account in order to know how the children respond to the intervention methodologies that are tested according to their degree of affectation as well as the quality of life perceived by the families. In addition, future research should focus on longitudinal and experimental studies to establish causal relationships between the implementation of specific intervention strategies and improvements in development and quality of life. In addition, these studies should seek to collect data on the medium- and long-term sustainability of intervention effects. Finally, it would be beneficial to explore the development of strategies that facilitate the inclusion of caregivers in the intervention process by the professional team.

## 5. Conclusions

Based on the results of our study, the use of the hybrid model, the family-centered model in combination with mHealth, in rural contexts appears to be a viable solution for improving access to early childhood intervention services. Teleintervention provide positive effects on both child development and family dynamics, enhancing satisfaction, confidence, and a sense of competence factors intrinsically linked to family quality of life.

Interdisciplinary approaches and individualized care are pivotal for establishing a strong therapeutic bond that maximizes the impact of these interventions and thus increasing the well-being of the family nucleus in the environment in which they live.

## Figures and Tables

**Table 1:** T-test differences between control group 1 and the experimental group.

	Pre-Test				Post-Test			
	F ^1^	gl	*p*	d	F ^1^	gl	*p*	d
BATTELLE	1.489	36	0.145	0.494	235.000 ^2^		0.013 *	0.729
PEDI Mobility	168.000 ^2^		0.938	−0.094	1.066	35	0.294	0.357
PEDI Social	1.196	34	0.240	0.404	0.834	35	0.410	0.279
FQL Total	0.856	32	0.398	0.302	116.500 ^2^		0.210	0.290
FQL RF	1.843	33	0.074	0.645	1.227	31	0.229	0.453
FQL AIS	0.891	33	0.379	0.312	0.027	32	0.979	0.010
FQL FN	0.014	32	0.989	0.005	−1.067	27	0.296	−0.408
ConFam Total	1.938	33	0.061	0.678	0.424	32	0.674	0.152
ConFan CAN	1.568	33	0.126	0.548	0.143	32	0.887	0.051
Confam CAST	2.041	33	0.049 *	0.714	0.766	32	0.449	0.275
FOS Total	0.871	33	0.390	0.305	0.840	32	0.407	0.302
FOS 1	140.500 ^2^		0.945	−0.017	−0.300	32	0.766	−0.108
FOS 2	2.023	33	0.051	0.708	1.398	32	0.172	0.502
FOS 3	139.500 ^2^		0.915	−0.024	−0.341	32	0.735	−0.122
FOS 4	0.227	33	0.822	0.079	1.371	32	0.180	0.492
FOS 5	175.000 ^2^		0.280	0.224	0.942	32	0.353	0.338

Note: ^1^ Student’s *t*-test; ^2^ Mann–Whitney U (non-parametric test—normality not assumed); df = degrees of freedom; * < 0.05; d = Cohen’s d.

**Table 2:** T-test differences between control group 2 and the experimental group.

	Pre-Test				Post-Test			
	F ^1^	gl	*p*	d	F ^1^	gl	*p*	d
BATTELLE	−2.534	31	0.017	−0.886	77.000 ^2^		0.037 *	−0.430
PEDI Mobility	−2.424 ^3^	30	0.022 *	−0.859	−2.270 ^3^	30	0.031 *	−0.804
PEDI Social	−1.168	30	0.252	−0.414	−1.701	30	0.099	−0.603
FQL Total	0.981	27	0.335	0.366	104.000 ^2^		0.113	0.387
FQL RF	2.782	27	0.010 *	1.039	2.391	25	0.025 *	0.936
FQL AIS	2.150	27	0.041 *	0.803	0.791	25	0.436	0.391
FQL FN	−1.014	27	0.320	0.378	84.000 ^2^		0.863	−0.045
ConFam Total	1.721	27	0.097	0.643	1.703	26	0.101	0.399
ConFan CAN	1.281	27	0.211	0.478	1.536	26	0.137	0.396
Confam CAST	1.907	27	0.067	0.712	1.555	26	0.132	0.396
FOS Total	1.158	27	0.257	0.432	1.309	26	0.202	0.392
FOS 1	−0.190	27	0.851	−0.071	0.942	26	0.355	0.387
FOS 2	141.000 ^2^		0.108	0.356	−0.035	26	0.972	0.382
FOS 3	110.500 ^2^		0.780	0.063	0.608	26	0.549	0.384
FOS 4	135.000 ^2^		0.175	0.298	2.612	26	0.015 *	0.997
FOS 5	132.000 ^2^		0.225	0.269	1.672	26	0.107	0.398

Note: ^1^ Student’s *t*-test; ^2^ Mann–Whitney U (non-parametric test—normality not assumed); ^3^ Welch’s *t*-test (non-parametric test—equality of variances not assumed);* < 0.05;d = Cohen’s d.

**Table 3:** Intra-group mean contrast tests between pre-test and post-test.

	Grupo Control 1					Grupo Control 2					Grupo Experimental				
	F ^1^	df	z	*p*	d ^3^	F ^1^	df	z	*p*	d ^3^	F ^1^	df	z	*p*	d ^3^
BATTELLE	1.000 ^2^		−3.883	<0.001 *	−0.990	−3.632	17		0.002 *	−0.856	51.000 ^2^		−0.511	0.629	−0.150
PEDI Mobility	18.500		−3.079	0.002 *	−0.805	−0.725	16		0.479	−0.176	−0.200	14		0.845	−0.052
PEDI Social	60.000		−1.111	0.276	−0.355	−1.480	16		0.158	−0.359	−2.745	14		0.016 *	−0.709
FQL Total	0.141	16		0.889	0.034	−0.742	14		0.470	−0.192	−0.684	9		0.511	−0.216
FQL RF	1.367	20		0.187	0.298	−0.397	15		0.697	−0.099	−0.760	10		0.465	−0.229
FQL AIS	−0.523	20		0.607	−0.114	0.111	14		0.914	0.029	−2.008	11		0.070 *	−0.580
FQL FN	84.000 ^2^		0.355	0.739	0.098	−1.358	15		0.195	−0.339	−0.419	10		0.684	−0.126
ConFam Total	0.929	20		0.364	0.203	−0.835	15		0.417	−0.209	−2.855	11		0.016 *	−0.824
ConFan CAN	0.801	20		0.433	0.175	−0.708	15		0.490	−0.177	−2.780	11		0.018 *	−0.802
Confam CAST	0.841	20		0.411	0.183	21.000 ^2^		−1.412	0.168	−0.462	−1.214	11		0.250	−0.350
FOS Total	0.996	20		0.331	0.217	0.811	15		0.430	0.203	1.894	11		0.085	0.547
FOS 1	3.154	20		0.005 *	0.688	0.216	15		0.832	0.054	2.244	11		0.046 *	0.648
FOS 2	−0.256	20		0.801	−0.056	37.500 ^2^		1.019	0.329	0.364	−2.714	11		0.020 *	−0.784
FOS 3	1.326	20		0.200	0.289	43.500 ^2^		1.631	0.111	0.582	1.890	11		0.085	0.546
FOS 4	−0.613	20		0.547	−0.134	31.000 ^2^		0.357	0.758	0.127	0.907	11		0.384	0.262
FOS 5	1.546	20		0.138	0.337	0.078	15		0.939	0.167	1.530	11		0.154	0.442

Note: ^1^ Student’s *t*-test; ^2^ Wilcoxon w-test; df = degrees of freedom; * < 0.05; ^3^ d = Cohen’s d.

## Data Availability

Data is unavailable due to privacy and ethical restrictions.

## Appendix A

**Table A1:** Shapiro–Wilk normality test for the three groups in pre-test and post-test.

	Grupo Control 1				Grupo Control 2				Grupo Experimental			
	Pre-Test		Post-Test		Pre-Test		Post-Test		Pre-Test		Post-Test	
	W	*p*	W	*p*	W	*p*	W	*p*	W	*p*	W	*p*
BATTELLE	0.956	0.394	0.959	0.498	0.952	0.458	0.959	0.590	0.933	0.307	0.858	0.022 *
PEDI Mobility	0.773	<0.001 *	0.621	<0.001	0.905	0.082	0.914	0.115	0.932	0.291	0.923	0.213
PEDI Social	0.970	0.725	0.948	0.289	0.931	0.227	0.944	0.374	0.940	0.386	0.915	0.162
FQL Total	0.936	0.181	0.974	0.869	0.919	0.164	0.977	0.944	0.952	0.622	0.706	0.001 *
FQL RF	0.973	0.769	0.926	0.099	0.938	0.326	0.956	0.594	0.939	0.439	0.865	0.068
FQL AIS	0.914	0.057	0.893	0.021	0.990	0.999	0.963	0.752	0.982	0.988	0.886	0.105
FQL FN	0.992	0.999	0.945	0.351	0.964	0.729	0.876	0.033 *	0.960	0.761	0.922	0.335
ConFam Total	0.989	0.995	0.984	0.970	0.966	0.772	0.971	0.862	0.947	0.559	0.916	0.255
ConFan CAN	0.968	0.674	0.987	0.990	0.937	0.312	0.973	0.884	0.981	0.982	0.987	0.999
Confam CAST	0.971	0.745	0.982	0.938	0.960	0.660	0.968	0.808	0.938	0.435	0.901	0.163
FOS Total	0.939	0.190	0.965	0.591	0.918	0.156	0.970	0.837	0.941	0.474	0.959	0.767
FOS 1	0.902	0.032 *	0.966	0.613	0.947	0.438	0.936	0.306	0.857	0.035 *	0.960	0.785
FOS 2	0.937	0.174	0.915	0.060	0.882	0.042 *	0.950	0.496	0.965	0.833	0.951	0.647
FOS 3	0.802	<0.001 *	0.959	0.462	0.811	0.004 *	0.938	0.329	0.810	0.009 *	0.811	0.013
FOS 4	0.915	0.060	0.877	0.010	0.803	0.003 *	0.910	0.115	0.891	0.100	0.900	0.158
FOS 5	0.910	0.047 *	0.912	0.051	0.860	0.019 *	0.867	0.024	0.901	0.138	0.984	0.994

Note: * < 0.05; *p*-values below 0.05 indicate a lack of normality.

**Table A2:** Levene’s test for homogeneity of variances in pre-test and post-test.

	Control Group 1 and Experimental Group								Control Group 2 and Experimental Group							
	Pre-Test				Post-Test				Pre-Test				Post-Test			
	F	df ^1^	df ^2^	*p*	F	df ^1^	df ^2^	*p*	F	df ^1^	df ^2^	*p*	F	df ^1^	df ^2^	*p*
BATTELLE	1.074	1	36	0.307	1.571	1	34	0.219	0.262	1	31	0.613	0.004	1	31	0.948
PEDI Mobility	0.267	1	35	0.609	0.191	1	35	0.665	12.593	1	30	0.001 *	7.026	1	30	0.013 *
PEDI Social	1.709	1	34	0.200	0.121	1	35	0.730	0.013	1	30	0.910	1.458	1	30	0.237
FQL Total	0.040	1	32	0.842	1.211	1	26	0.281	0.877	1	27	0.357	1.185	1	23	0.288
FQL RF	0.367	1	33	0.549	1.554	1	31	0.222	0.348	1	27	0.560	2.345	1	25	0.138
FQL AIS	0.047	1	33	0.830	0.547	1	32	0.465	1.271	1	27	0.270	0.344	1	25	0.563
FQL FN	0.208	1	32	0.651	0.089	1	27	0.768	1.628	1	27	0.213	0.061	1	25	0.806
ConFam Total	0.050	1	33	0.825	6.219 × 10^−4^	1	32	0.980	0.532	1	27	0.472	0.048	1	26	0.828
ConFan CAN	1.473	1	33	0.233	0.174	1	32	0.680	0.095	1	27	0.760	0.040	1	26	0.844
Confam CAST	0.001	1	33	0.975	0.184	1	32	0.671	0.225	1	27	0.639	0.651	1	26	0.427
FOS Total	0.006	1	33	0.937	0.276	1	32	0.603	0.941	1	27	0.341	0.390	1	26	0.538
FOS 1	0.178	1	33	0.676	0.099	1	32	0.755	0.004	1	27	0.951	0.231	1	26	0.635
FOS 2	0.365	1	33	0.550	0.027	1	32	0.870	3.129	1	27	0.088	0.040	1	26	0.843
FOS 3	0.012	1	33	0.915	0.210	1	32	0.650	0.205	1	27	0.655	1.106	1	26	0.303
FOS 4	0.009	1	33	0.926	0.183	1	32	0.672	0.449	1	27	0.509	1.275	1	26	0.269
FOS 5	0.037	1	33	0.848	0.675	1	32	0.417	0.838	1	27	0.368	0.262	1	26	0.613

Note: ^1^ Student’s *t*-test; ^2^ Mann–Whitney U (non-parametric test—normality not assumed); df = degrees of freedom; * < 0.05; *p*-values below 0.05 indicate a lack of homogeneity of variances.
